# *Monascus*-Fermented Dioscorea Enhances Oxidative Stress Resistance via DAF-16/FOXO in *Caenorhabditis elegans*

**DOI:** 10.1371/journal.pone.0039515

**Published:** 2012-06-22

**Authors:** Yeu-Ching Shi, Chan-Wei Yu, Vivian Hsiu-Chuan Liao, Tzu-Ming Pan

**Affiliations:** 1 Department of Bioenvironmental Systems Engineering, National Taiwan University, Taipei, Taiwan; 2 Department of Biochemical Science and Technology, National Taiwan University, Taipei, Taiwan; University of Birmingham, United Kingdom

## Abstract

**Background:**

*Monascus*-fermented products are mentioned in an ancient Chinese pharmacopoeia of medicinal food and herbs. *Monascus*-fermented products offer valuable therapeutic benefits and have been extensively used in East Asia for several centuries. Several biological activities of *Monascus*-fermented products were recently described, and the extract of *Monascus*-fermented products showed strong antioxidant activity of scavenging DPPH radicals. To evaluate whether *Monascus*-fermented dioscorea products have potential as nutritional supplements, *Monascus*-fermented dioscorea’s modulation of oxidative-stress resistance and associated regulatory mechanisms in *Caenorhabditis elegans* were investigated.

**Principal Findings:**

We examined oxidative stress resistance of the ethanol extract of red mold dioscorea (RMDE) in *C. elegans*, and found that RMDE-treated wild-type *C. elegans* showed an increased survival during juglone-induced oxidative stress compared to untreated controls, whereas the antioxidant phenotype was absent from a *daf-16* mutant. In addition, the RMDE reduced the level of intracellular reactive oxygen species in *C. elegans*. Finally, the RMDE affected the subcellular distribution of the FOXO transcription factor, DAF-16, in *C. elegans* and induced the expression of the *sod-3* antioxidative gene.

**Conclusions:**

These findings suggest that the RMDE acts as an antioxidative stress agent and thus may have potential as a nutritional supplement. Further studies in *C. elegans* suggest that the antioxidant effect of RMDE is mediated via regulation of the DAF-16/FOXO-dependent pathway.

## Introduction

Red mold rice has long been used as a traditional food and dairy supplement. It was mentioned in an ancient Chinese pharmacopoeia of medicinal food and herbs. Several biological activities of red mold rice were recently described, including inhibition of the biosynthesis of cholesterol for treating hyperlipidemia [1], improvements of the memory and learning ability in Aβ-infused rats, and a reduction in blood glucose levels in diabetic rats [2], [3]. In recent years, red mold dioscorea has been extensively studied. Red mold dioscorea contains various metabolites, including dimerumic acid, tannins, phenols, and polyketides (monacolins) with antioxidative properties and anti-inflammatory responses [4], [5]. In particular, monacolins are believed to account for the majority of the cholesterol-controlling activity of *Monascus* [1]. An extract of *M. anka* was shown to exhibit strong antioxidant action of scavenging the 1-1-diphenyl-2-picrylhydrazyl (DPPH) radical and inhibiting lipid peroxidation [6]. In our previous study, the ethanol extract of red mold rice (RMRE) was shown to have antioxidative activity of scavenging DPPH radicals [7]. In addition, the constituents of the RMDE were complicated with monascin and ankaflavin being the major components [8].

Oxidative stress occurs in cells with excessive production of reactive oxygen species (ROS) such as a superoxide anion (O_2_^-^) and hydrogen peroxide (H_2_O_2_) which can overwhelm a cell’s natural antioxidant defense, leading to injury to biomolecules such as lipids, proteins, and DNA [9]. Oxidative stress is thought to contribute to the general decline in cellular functions associated with human diseases, such as Alzheimer’s disease [10], [11], atherosclerosis [12], diabetes [13], [14], Parkinson’s disease [15], [16], and human cancers [17], [18] as well as the aging [19], [20] process itself.

The nematode *Caenorhabditis elegans* (*C. elegans*) has become a popular model to study molecular mechanisms of drug effects and disease pathogeneses. Many key findings with relevance for mammals were discovered in the well-characterized *C. elegans*. There is strong conservation of biological principles between *C. elegans* and mammals, and 60%∼80% of human gene homologues have been identified in *C. elegans* [21]. *C. elegans* has been used to study various age or oxidative stress-associated diseases, including Alzheimer’s [22], [23] and diabetes [24], [25]. In contrast to cell-culture systems and animal experiments, *C. elegans* is easy to culture, and because of the transparent appearance of the worms, fluorescent markers, such as reporter genes, can be observed in living animals [26]. The insulin/insulin-like growth factor (IGF)-1 signal transduction pathway plays an important role in regulating both longevity and stress resistance [27]. Activity of DAF-16, a FOXO transcription factor, is influenced by the insulin pathway which is involved in antioxidative defense, stress resistance, and metabolism [28].

In order to evaluate whether *Monascus*-fermented dioscorea products have potential as nutritional supplements, modulation of the *in vivo* antioxidant activity of the ethanol extract of red mold dioscorea (RMDE) and its associated regulatory mechanism in *C. elegans* were investigated. Herein, we analyzed the antioxidant activity of the RMDE by oxidative-stress assays and measured intracellular ROS levels in *C. elegans*. In addition, factors and genetic requirements that influence oxidative-stress resistance by the RMDE are dissected.

## Results

### The RMDE Enhanced Oxidative-stress Resistance in *C. elegans*

**Figure 1:**
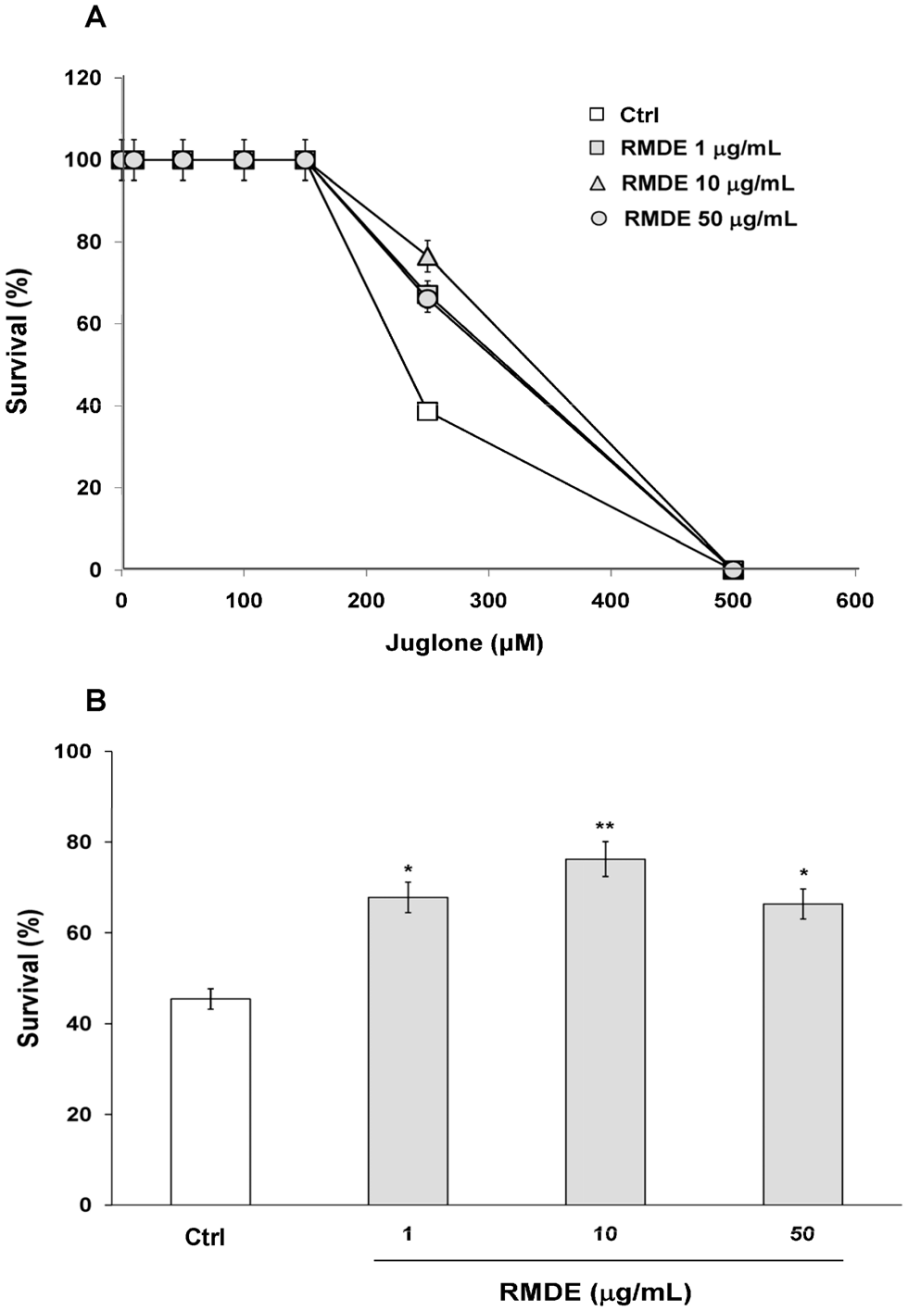
Effects of the ethanol extract of red mold dioscorea (RMDE) on oxidative-stress resistance of wild-type (WT) *Caenorhabditis elegans* N2. (A) Dose response for juglone exposure. Synchronized WT L1 larvae were pretreated with the RMDE (1, 10, and 50 µg/mL) or 0.1% DMSO as the solvent control for 72 h at 20°C. Subsequently, adult worms were subjected to oxidative-stress assays. For the oxidative-stress assays, RMDE-treated (*n = *100) and control (0.1% DMSO, *n = *100) adult worms were exposed to 0, 10, 50, 100, 150, 150, 250, and 500 µM juglone for 3.5 h at 20°C and then scored for viability. (B) Survival of RMDE-treated and control adult worms exposed to 250 µM juglone for 3.5 h at 20°C. The test was performed three times. Error bars represent the standard error, and differences compared to the control (0 µg/mL, 0.1% DMSO) were considered significant at *p*<0.05 (*), *p*<0.01 (**) by one-way ANOVA and the LSD post-hoc test.

The RMDE was prepared and analyzed as described previously [8]. To investigate whether RMDE has an antioxidant effect in *C. elegans*, wild-type (WT) N2 worms were pretreated with RMDE, followed by exposure to juglone-induced oxidative stresses. WT N2 synchronized L1 larvae were pretreated with 1, 10, and 50 µg/mL RMDE and 0.1% DMSO as solvent control for 72 h at 20°C. Adult animals were then exposed to 0, 10, 50, 100, 150, 150, 250, and 500 µM juglone, a redox cycler that generates intracellular oxidative stress [29] and then they were incubated for 3.5 h (Fig. 1A).

During pretreatment with the RMDE, no adverse effects on the worms, including survival, growth rate, progeny production, body length, or morphological changes, were observed. The dose response analysis for juglone showed that 250 µM juglone was the optimal concentration to examine the antioxidant effect of RMDE in *C. elegans* (Fig. 1A). The results showed that pretreatment with 1, 10, and 50 µg/mL RMDE significantly increased the survival of worms exposed to juglone-induced oxidative stress (Fig. 1A, B).

The oxidative stress assays were further evaluated by using H_2_O_2_ and paraquat. After RMDE (10 µg/mL) treatment, adult WT N2 worms were exposed to 1 mM H_2_O_2_ [30]–[32] and 150 mM paraquat [33]–[35] for 4.5 h and 12 h, respectively and then the viability of worms was scored. In agreement with the observation by juglone, RMDE (10 µg/mL) significantly increased the survival of WT N2 worms upon H_2_O_2_ and paraquat exposure (Fig. S1).

### The RMDE Decreased Intracellular ROS Levels in *C. elegans*

**Figure 2:**
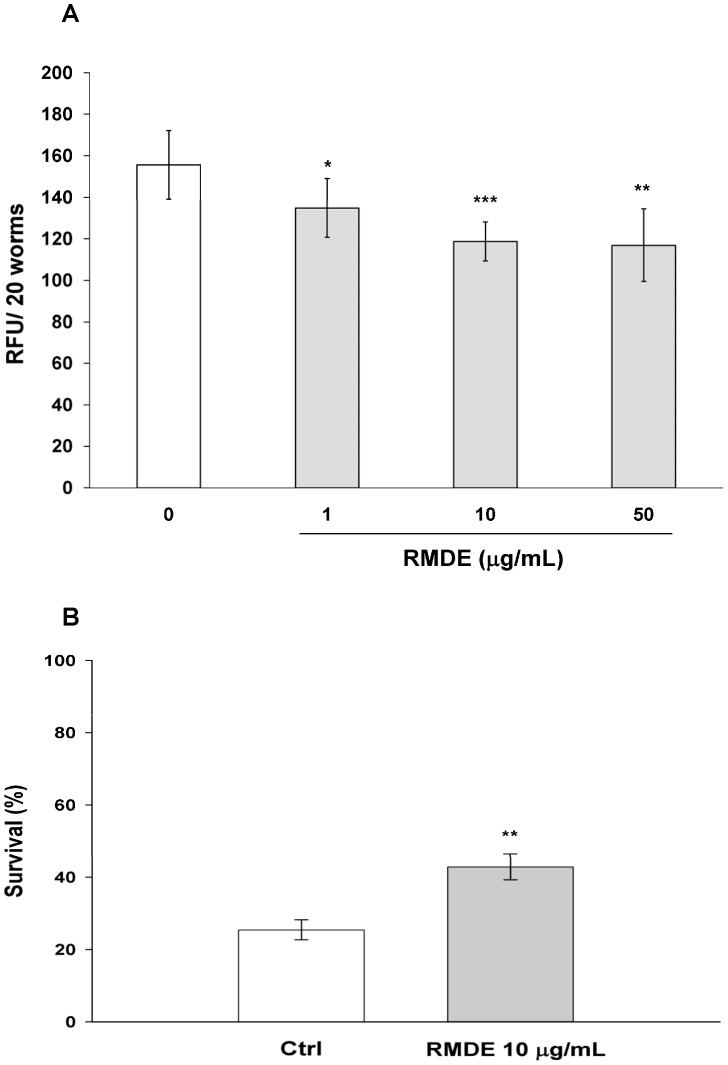
Effects of the ethanol extract of red mold dioscorea (RMDE) on reactive oxygen species (ROS) accumulation in *Caenorhabditis elegans*. (A) Synchronized WT L1 larvae were pretreated with the RMDE or 0.1% DMSO as the solvent control for 72 h at 20°C. Subsequently, intracellular ROS were analyzed and the results are presented as relative fluorescence units (RFU) of 20 individual worms (*n = *160). (B) Oxidative-stress resistance of the *mev-1* mutant. Synchronized *mev-1* L1 larvae were pretreated with the RMDE or 0.1% DMSO as the solvent control for 72 h at 20°C. Subsequently, mutant worms were subjected to oxidative-stress assays. RMDE-treated (*n = *76) and control (0.1% DMSO, *n = *101) worms were exposed to 250 µM juglone for 3.5 h at 20°C and then scored for viability. The test was performed three times. Error bars represent the standard error, and differences compared to the control (0 µg/mL, 0.1% DMSO) were considered significant at *p*<0.05 (*), *p*<0.01 (**), and *p*<0.001 (***) by one-way ANOVA and the LSD post-hoc test.

Next, we examined whether RMDE-enhanced oxidative-stress resistance was due to its ROS-scavenging ability. Non-fluorescent DCF-DA is a freely cell-permeable dye, which is readily converted to the fluorescent 2′7′-dichlorofluorescein (DCF) due to an interaction with intracellular H_2_O_2_. Figure 2A shows that the RMDE significantly decreased intracellular ROS when WT N2 worms were treated with 1, 10, and 50 µg/mL RMDE. Since as low as 10 µg/mL RMDE was able to significantly alleviate the amount of intracellular ROS as well as significantly enhance the antioxidant effect, 10 µg/mL RMDE was chosen as the working concentration for further experiments.

We next examined RMDE’s ROS-scavenging ability using the *mev-1* mutant. *mev-1* is a defect of the mitochondrial complex which exhibits increased ROS levels *in vivo*. Thus, *mev-1* mutant is hypersensitive to elevated oxygen levels [36]. After RMDE treatment, adult *mev-1* animals were exposed to 250 µM juglone for 3.5 h. The survival of *mev-1* mutants significantly increased with 10 µg/mL RMDE treatment compared to that of untreated ones (Fig. 2B). Taken together, RMDE may act against oxidative stress through its intracellular ROS-scavenging ability and decreases mitochondrial ROS toxicity.

### The RMDE Enhanced Oxidative-stress Resistance in *C. elegans* Via DAF-16

**Figure 3:**
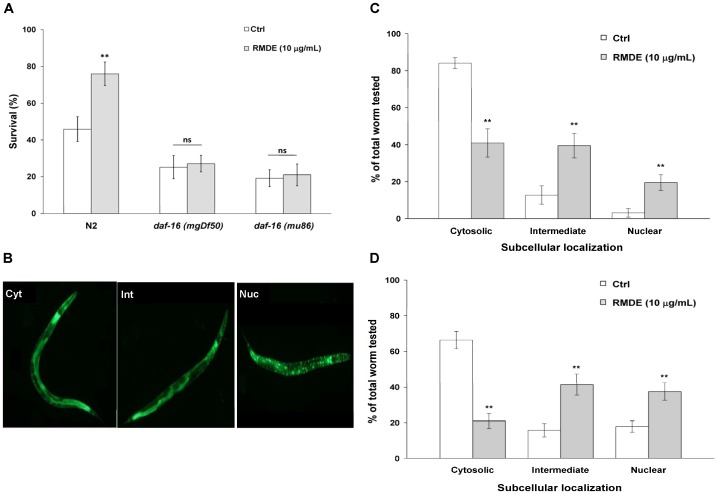
Effects of the ethanol extract of red mold dioscorea (RMDE) on DAF-16. (A) Survival of *daf-16* deletion mutants under oxidative stress. Synchronized L1 larvae were pretreated with the RMDE (10 µg/mL, WT N2. *n = *100; *daf-16(mgDf50)*, *n* = 98; *daf-16(mu86)*, *n* = 120) or 0.1% DMSO as the solvent control (WT N2, *n = *100; *daf-16(mgDf50)*, *n* = 108; *daf-16(mu86)*, *n* = 143) for 72 h at 20°C. Subsequently, worms were subjected to oxidative-stress assays. Worms were exposed to 250 µM juglone for 3.5 h at 20°C and then scored for viability. (B) Effect of RMDE on subcellular DAF-16 localization. Immediately after hatching, age-synchronized transgenic *C. elegans* L1 larvae (TJ356) were pretreated with the RMDE (10 µg/mL) and 0.1% DMSO as the solvent control for 72 h at 20°C. The subcellular distribution of DAF-16 was examined in approximately 20 animals per condition by fluorescence microscopy. Worms were classified into the categories of “cytosolic” (left panel), “intermediate” (middle panel), and “nuclear” (right panel) according to their localization phenotypes. Comparative evaluation of the subcellular distribution of DAF-16 in the groups (C) without and (D) with oxidative stress. For the oxidative-stress assays, RMDE-treated (10 µg/mL) and control (0.1% DMSO) adult worms were challenged with 50 µM juglone for 5 min, and then their fluorescence intensity was scored. The results are presented as the percentages of each localization phenotype in the total worms tested. The test was performed three times. Error bars represent the standard error, and differences compared to the control (0.1% DMSO) were considered significant at *p*<0.01 (**) by one-way ANOVA and the LSD post-hoc test. ns, no significant.

To identify RMDE’s mechanism of action, we examined its effect on DAF-16. DAF-16 is a forkhead (FOXO) transcription factor with crucial functions in controlling stress responses and aging in *C. elegans* and mammals [37]. To analyze the involvement of DAF-16 in RMDE-mediated oxidative-stress resistance, we performed a juglone-induced oxidative stress test with *daf-16(mgDf50)* and *daf-16(mu86)* deletion mutants. Both *daf-16(mgDf50)* and *daf-16(mu86)* are null mutants [38], [39]. For the 250 µM juglone-induced oxidative stress tests, unlike WT N2 worms, the survival of both RMDE-treated *daf-16(mgDf50)* and *daf-16(mu86)* mutants did not show a significant difference compared to that of untreated ones (Fig. 3A).

Since the *daf-16* mutant is more sensitive to juglone, we further evaluated how the *daf-16* mutant behaves at a lower juglone concentration, and whether RMDE has an effect at this lower concentration. We used 150 µM juglone at which WT N2 worms did not show significantly different survival between RMDE untreated and treated conditions (Fig. 1A) to evaluate the effect of RMDE on *daf-16* mutant. The result showed that survival of RMDE treated and untreated control *daf-16(mu86)* mutant was not significantly different upon 150 µM juglone exposure (Figure S2). This is in agreement with the effects of RMDE on *daf-16(mgDf50)* and *daf-16(mu86)* mutants by using higher concentration juglone (250 µM) (Fig. 3A, Fig. S2). Taken together, the RMDE may provide oxidative-stress resistance in *C. elegans* via DAF-16.

To further investigate the role of DAF-16 in modulating RMDE-induced oxidative-stress resistance, we examined the translocation of DAF-16. Localization of DAF-16 in nuclei is an essential prerequisite for its ability to activate target gene transcription [40]–[43]. Localization of DAF-16 was studied under normal culture conditions and in cultures exposed to juglone. The subcellular distribution of DAF-16 was classified into the categories of “cytosolic”, “intermediate”, and “nuclear” according to their localization phenotypes (Fig. 3B). Control untreated worms incubated under normal culture conditions revealed a predominant cytosolic localization of DAF-16 (Fig. 3 C). Treatment of transgenic worms with 10 µg/mL RMDE for 3 days resulted in increases in the fractions of worms showing nuclear localization of DAF-16 with cytosolic (41.0% ±7.7%), intermediate (39.5% ±6.6%), and nuclear (19.5% ±4.3%) localization phenotypes (Fig. 3C). Following exposure to 50 µM juglone for 5 min, fractions of intermediate and nuclear localization of DAF-16 were greater in RMDE-treated transgenic worms (Fig. 3D). Therefore, RMDE affected the subcellular distribution of DAF-16 and caused translocation of DAF-16 from the cytoplasm to nuclei. Taken together, the results suggest that RMDE enhanced oxidative-stress resistance in *C. elegans* via DAF-16.

### The RMDE Upregulated SOD-3 Expression

To elucidate whether the above-described increase in oxidative-stress resistance was due to the RMDE regulating a specific stress-response gene, we examined the responsiveness of the antioxidant enzyme, SOD, to RMDE treatment. SOD is a major enzyme that protects against oxidative stress by catalyzing the removal of O_2_^-^ [44]. In *C. elegans*, *sod-3* encodes manganese (Mn)SOD, and *sod-3* is known to be a target gene of DAF-16 [37].

**Figure 4:**
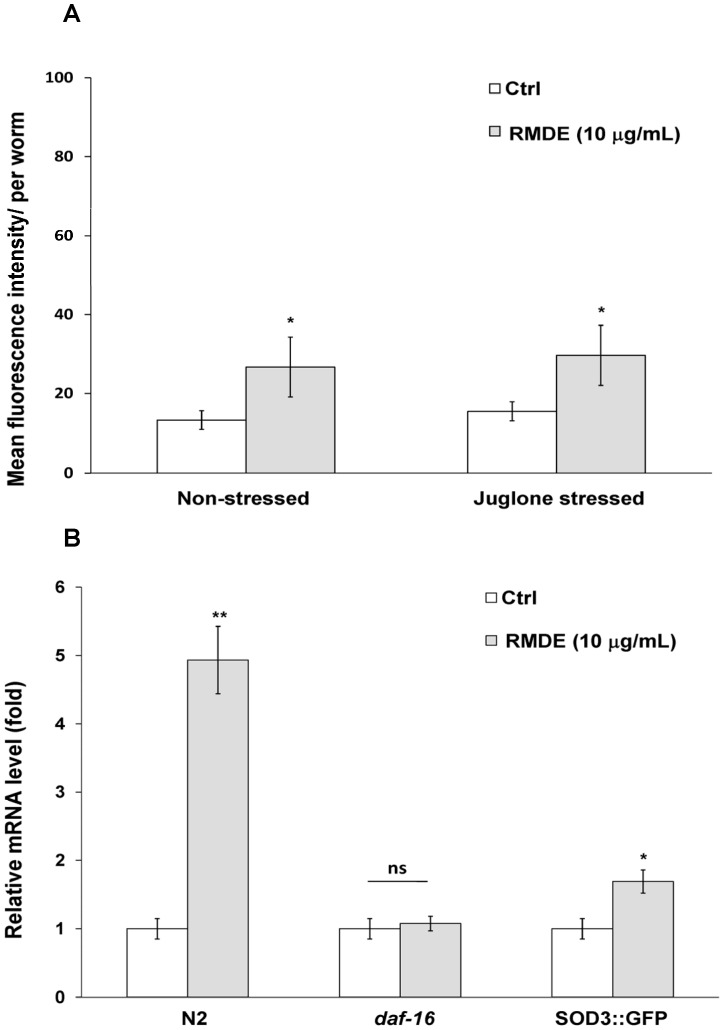
Effects of the ethanol extract of red mold dioscorea (RMDE) on the expression of superoxide dismutase (SOD). (A). Effects of RMDE on the expression of SOD-3::green fluorescent protein (GFP). Immediately after hatching, age-synchronized, transgenic L1 worms of the CF1553 strain (*sod-3*:: GFP) were treated with 10 µg/mL RMDE or 0.1% DMSO as the solvent control for 72 h at 20°C, followed by 150 µM juglone exposure for 1 h. Subsequently, the total GFP fluorescence of each whole worm was quantified by Image-Pro Plus software. Data shown are the average number of pixels in transgenic *Caenorhabditis elegans* (*n* = 20) at each indicated treatment. (B) Effects of RMDE on the SOD-3 mRNA level. Synchronized WT N2, *daf-16(mgDf50)* mutant, and CF1553 strain L1 larvae were pretreated with 10 µg/mL RMDE or 0.1% DMSO as the solvent control for 72 h at 20°C and then total RNA was extracted. mRNA level of SOD-3 was determined by quantitative real-time RT-PCR. SOD-3 mRNA levels were normalized to the expression of AMA-1. The fold change was normalized to that observed in untreated control *C. elegans* samples. The test was performed three times. Error bars represent the standard error, and differences compared to the control (0.1% DMSO) were considered significant at *p*<0.05 (*), *p*<0.01 (**) by one-way ANOVA and the LSD post-hoc test. ns, no significant.

The CF1553 transgenic strain which contains a reporter gene of *sod-3* fused to GFP was treated with 10 µg/mL RMDE for 3 days. Figure 4A shows that 10 µg/mL RMDE treatment alone significantly upregulated SOD-3::GFP expression compared to untreated worms. Additionally, when strain CF1553 was further challenged with 150 µM juglone exposure for 1 h, the results showed that the expression level of SOD-3::GFP was also significantly increased in CF1553 worms treated with RMDE compared to that untreated ones (Fig. 4A).

To further confirm SOD-3 expression is regulated by RMDE, we measured SOD-3 mRNA by qRT-PCR (Fig. 4B). In addition to examine SOD-3 mRNA level in WT N2 nematodes in response to RMDE, SOD-3 mRNA levels in response to RMDE were also investigated in *daf-16(mgDf50)* mutant and CF1553 strain. The results showed that RMDE caused a 4.9-fold and 1.7-fold increase of SOD-3 mRNA level in WT N2 and CF1553, respectively comparing with that untreated ones (Fig. 4B). In contrast, SOD-3 mRNA level was diminished in *daf-16(mgDf50)* mutant showing no significant difference between RMDE-treated and untreated worms (Fig. 4B). Therefore, the results further confirmed the involvement of SOD-3 expression and DAF-16 in regulating RMDE antioxidant properties. Taken together, these findings suggest that RMDE may act against oxidative stress via DAF-16/FOXO.

## Discussion

The RMDE was studied *in vitro* and *in vivo*, and several biological effects were reported in mammalian systems [4], [5], [45]. However, its potential as a nutritional supplement and the mechanisms by which it exerts its action *in vivo* still remain to be further elucidated. Herein, we used *C. elegans* as an *in vivo* model to examine the protective potential and mode of action of the RMDE. There are several studies in which the protective actions of flavonoids and polyphenols in *C. elegans* were mainly attributed to their antioxidative activities [26], [46]. In the present study, we showed that the survival of WT worms was significantly increased with RMDE treatment under oxidative stress agents (juglone, H_2_O_2_, and paraquat)-induced oxidative stress (Fig. 1, Fig. S1), suggesting that the RMDE has antioxidative activity in these worms. An important feature of the toxicity of oxidative stress is presumably the elevated generation of ROS. Results showed that the RMDE significantly decreased intracellular levels of ROS within WT *C. elegans* (Fig. 2A). Furthermore, the RMDE enhanced the survival of the *mev-1* mutant (Fig. 2B) which is an ROS-generating strain. The results demonstrated that the RMDE may act against oxidative stress due to its ROS-scavenging ability, which decreased mitochondrial ROS toxicity.

To identify RMDE’s mechanism of action, we examined its effect on DAF-16/FOXO. FOXO transcription factors are conserved from worms to human and regulated by insulin signaling pathway [37]. DAF-16 is the only FOXO protein in *C. elegans* [37]. In *C. elegans*, the DAF-16 transcription factor in the insulin signaling pathway is considered a key regulator of many important biological processes including lifespan, metabolism, and stress responses [47]. We examined the ability of loss-of-function *daf-16* deletion mutants to resist oxidative stress during RMDE exposure. The premise was that if *daf-16* gene is required for oxidative-stress resistance by the RMDE, then the RMDE would be unable to enhance oxidative-stress resistance in that mutant. Results showed that *daf-16* null mutants did not exhibit a significantly enhanced survival after RMDE treatment followed by juglone exposure compared to untreated worms (Fig. 3A). This indicated that the RMDE mediates oxidative-stress resistance via DAF-16.

To further validate that DAF-16 is required for the protective effect of the RMDE, the effect of the RMDE on the translocation of DAF-16 from the cytoplasm to nuclei was examined (Fig. 3C, D). In addition, the gene expression of SOD-3, a target gene of DAF-16, was examined after RMDE exposure (Fig. 4A, B). Results showed that the RMDE was able to enhance DAF-16 translocation from the cytoplasm to nuclei (Fig. 3C, D). Moreover, SOD-3 expression was induced by RMDE exposure (Fig. 4A). Furthermore, RMDE caused a significant increase of SOD-3 mRNA level in WT N2 and CF1553 comparing with the untreated ones (Fig. 4B). In contrast, SOD-3 mRNA level was diminished in *daf-16* mutant showing no significant difference between RMDE-treated and untreated worms (Fig. 4B). Nuclear localization of DAF-16 is a prerequisite for transcriptional activation of its target genes such as genes for antioxidative enzymes like MnSOD (*sod*-3) and catalases (*ctl*-1 and *ctl*-2) [28]. Therefore, the antioxidant effect of the RMDE is likely mediated via regulation of a DAF-16-dependent pathway. It is noted that there are conflicting data regarding the response of SOD-3 to juglone exposure. Van Raamsdonk and Hekimi [48] found that a *sod-3* deletion does not affect juglone resistance significantly. They found a small effect with paraquat but this is not found by Doonan et al [49]. In contrast, Heidler et al [50] showed that SOD-3 expression and enzymatic activity were increased upon juglone exposure. Therefore, although our results showed that RMDE enhances SOD-3 upregulation and support the activation of DAF-16 by RMDE, it is not necessary that SOD-3 mediates the stress resistance as other DAF-16 target genes might also involved in RMDE-induced resistance.

RMDE itself can lead to nuclear localization of DAF-16 (Fig. 3C) and induce expressions of SOD-3 in transgenic strain carrying SOD-3::GFP (Fig. 4A) and mRNA level (Fig. 4B). This suggests that RMDE has similar effect as mild stress stimulus. In spite of the deleterious effects of ROS, recent research has indicated that ROS serve many important and non-damaging roles in both intracellular and extracellular signal transduction that involves diverse functions from vascular health to host defense [51]. Stress hormesis occurs when a low level stress elicits adaptive beneficial responses that protect against subsequent exposure to severe stress [52]. Recent findings suggest that mild oxidative stress from low concentrations of juglone can extend *C. elegans* lifespan by hormetic mechanisms [50]. Therefore, it is possible that by acting as mild stress stimulus, RMDE activates an adaptive response leading to an increase resistance in *C. elegans* to a subsequent severe stress (such as high concentration, 250 µM juglone exposure).

In conclusion, our study is the first to demonstrate that the RMDE has the capacity to increase *in vivo* oxidative-stress resistance. This finding is supported by recent studies showing that the RMDE exerts protection against oxidative stress and diabetic disease-associated inflammation [3], [4], [7], [45], [53]. Further study in *C. elegans* suggests that the antioxidative effect of the RMDE is mediated via regulation of DAF-16/FOXO-dependent pathway. These findings indicate that the RMDE acts as an antioxidative stress agent and thus may have potential as a nutritional supplement. Additionally, the RMDE may have therapeutic potential for preventing oxidative stress-associated diseases like diabetes and Alzheimer’s disease as well as aging.

## Materials and Methods

### *Caenorhabditis Elegans* Strains and Handling Procedures

Strains used in this study were: Bristol N2 (wild-type; WT); GR1307, *daf-16(mgDf50)*; CF1038, *daf-16(mu86)*; TK22, *mev-1* (*kn1*); CF1553, muIs84[pAD76(*sod-3*::GFP)], and TJ356, zIs356[DAF-16::GFP]. All *C. elegans* strains as well as the *Escherichia coli* OP50 strain were obtained from the *Caenorhabditis* Genetics Center (CGC), University of Minnesota, MN, USA). Worms were maintained (unless otherwise stated) at 20°C on nematode growth medium (NGM) agar plates carrying a lawn of *E. coli* OP50 according to Brenner [54]. Synchronization of worm cultures was achieved by hypochlorite treatment of gravid hermaphrodites [55].

### Stress-resistance Assays

Synchronized WT or mutant strain L1 larvae were incubated in liquid S-basal containing *E. coli* OP50 bacteria at 10^9^ cells/mL and various concentrations of the RMDE or 0.1% dimethyl sulfoxide (DMSO) as the solvent control for 72 h. Subsequently, adult worms were subjected to oxidative-stress assays. For the oxidative-stress assays, 5-hydroxy-1,4-naphthoquinone (juglone), hydrogen peroxide (H_2_O_2_), and paraquat (Sigma, St. Louis, MO, USA) were used to induce oxidative stress in worms. RMDE-treated and control adult worms were transferred to S-basal containing various concentrations of juglone, incubated for 3.5 h at 20°C, and then scored for viability. The survival of worms was determined by touch-provoked movement. Worms were scored as dead when they failed to respond to repeated touching with a platinum wire pick. The test was performed at least three times.

### Measurement of ROS

Intracellular ROS in *C. elegans* were measured using 2′,7′-dichlorodihydrofluoroscein diacetate (H_2_DCFDA) (Sigma). Synchronized WT L1 larvae were incubated in liquid S-basal containing *E. coli* OP50 bacteria at 10^9^ cells/mL and various concentrations of the RMDE or 0.1% DMSO as the solvent control for 72 h. The adult worms were then washed three times with phosphate buffered saline (PBS), followed by incubation in 250 µL PBS containing 100 µM H_2_DCFDA for 3 h. Subsequently, worms were washed twice with PBS and individual worms of each population were transferred into the wells of a 96-well microtiter plate containing 100 µL PBS. The ROS measurement was carried out in an FLx800 Microplate Fluorescent Reader (Bio-Tek Instruments, Winookski, VT, USA) for quantification of fluorescence with excitation at 485 nm and emission at 530 nm. The results are presented as relative fluorescence units (RFU) of 20 individual worms. The test was performed at least three times.

### Induction of a Stress-response Reporter

Synchronized L1 larvae containing an inducible green fluorescent protein (GFP) reporter for *sod-3*::GFP were incubated in liquid S-basal containing *E. coli* OP50 bacteria at 10^9^ cells/mL and a final concentration of 10 µg/mL RMDE or 0.1% DMSO as solvent control for 72 h and the expression of *sod-3*::GFP was measured. For juglone stressed assay, adult worms were then incubated for 1 h in S-basal containing 150 µM juglone [56]. The expression of *sod-3*::GFP was directly measured by observing the fluorescence of the GFP reporter protein. Twenty randomly selected worms from each set of experiments were mounted onto microscope slides coated with 2% agarose, anaesthetized with 2% sodium azide, and capped with coverslips. Epifluorescence images were captured with a Leica epifluorescence microscope (Leica, Wetzlar, Germany) using a suited filter set (with excitation at 480±20 nm and emission at 510±20 nm) with a cooled charge-coupled device (CCD) camera. Adult worms were examined, and total GFP fluorescence for each whole worm was quantified by Image-Pro Plus software (Media Cybernetics, Bethesda, MD, USA). The test was performed at least three times.

### Subcellular DAF-16 Localization

Synchronized L1 larvae of the TJ356 transgenic strain stably expressing a DAF-16::GFP fusion protein as a reporter [37] were incubated in liquid S-basal containing *E. coli* OP50 bacteria at 10^9^ cells/mL and a final concentration of 10 µg/mL RMDE or 0.1% DMSO as the solvent control for 72 h. The effect of the RMDE on oxidative stress was also examined by treatment with 50 µM juglone for 5 min. Subsequent to this treatment, worms were placed on microscope slides and capped with coverslips, and the subcellular DAF-16 distribution was analyzed by fluorescence microscopy on an epifluorescence microscope (Leica). Expression patterns of TJ356 worms were classified into three categories (cytosolic, intermediate, and nuclear) with respect to major localization of the DAF-16::GFP fusion protein. Subcellular DAF-16 localization was examined in approximately 20 animals per condition. The test was performed at least three times.

### RNA and Real-time Quantitative Reverse-transcription Polymerase Chain Reaction (qRT-PCR) Analysis

Synchronized L1 larvae were incubated in liquid S-basal containing *E. coli* OP50 bacteria at 10^9^ cells/mL and a final concentration of 10 µg/mL RMDE or 0.1% DMSO as solvent control for 72 h. Total RNA from adult worms was isolated using TRIzol according to manufacturer’s instructions (Invitrogen, Carlsbad, CA, USA) and cDNA was synthesized using SuperScript III First-strand synthesis super-Mix for qRT-PCR (Invitrogen). The qRT-PCR was performed on a Plus One real-time cycler (Applied Biosystems, Carlsbad, CA, USA) using a SYBR Green PCR core kit (Applied Biosystems). RT-PCR levels were normalized to the expression of *ama-1*, which encodes the large subunit of RNA polymerase II [57], [58]. The fold change was normalized to that observed in untreated *C. elegans* samples. The qRT-PCR primers for AMA-1 are: forward primer: 5′- CTGACCCAAAGAACACGGTGA-3′; reverse primer: 5′-TCCAATTCGATCCGAAGAAGC-3′. Primers for SOD-3 are: forward primer: 5′-AGCATCATGCCACCTACGTGA-3′; reverse primer: 5′-CACCACCATTGAATTTCAGCG-3′. The test was performed three times.

### Statistical Analyses

Statistical analyses were performed using SAS® 9.2 Software (SAS Institute, Cary, NC, USA). Results are presented as the mean ± standard error. The statistical significance of differences between populations was demonstrated by a one-way analysis of variance (ANOVA) and least significant difference (LSD) post-hoc test. Differences were considered significant at *p*<0.05, *p*<0.01, or *p*<0.001 (see Figures).

## Supporting Information

Figure S1 **Effects of the ethanol extract of red mold dioscorea (RMDE) on oxidative-stress resistance of wild-type (WT)** ***Caenorhabditis elegans*** **N2 using different oxidative stress agents.** Synchronized WT L1 larvae were pretreated with the RMDE 10 µg/mL or 0.1% DMSO as the solvent control for 72 h at 20°C. Subsequently, adult worms were subjected to oxidative-stress assays. Worms used for oxidative-stress assays were: RMDE-treated (juglone, *n* = 100; H_2_O_2_, *n* = 75; paraquat, *n* = 45) and 0.1% DMSO control (juglone, *n* = 100; H_2_O_2_, *n* = 75; paraquat, *n* = 45). Adult worms were exposed to 250 µM juglone, 1 mM H_2_O_2_, and 150 mM paraquat for 3.5 h, 4.5 h, and 12 h at 20°C, respectively and then scored for viability. The test was performed three times. Error bars represent the standard error, and differences compared to the control (0 µg/mL, 0.1% DMSO) were considered significant at *p*<0.05 (*), *p*<0.01 (**) by one-way ANOVA and the LSD post-hoc test.
(TIF)

Figure S2 **Effects of the ethanol extract of red mold dioscorea (RMDE) on DAF-16 by using different concentrations of juglone.** Synchronized *daf-16 (mu86)* mutant L1 larvae were pretreated with the RMDE (10 µg/mL) (150 µM juglone, *n* = 116; 250 µM juglone, *n* = 118) or 0.1% DMSO as the solvent control (150 µM juglone, *n* = 111; 250 µM juglone, *n* = 143) for 72 h at 20°C. Subsequently, worms were subjected to oxidative-stress assays. RMDE-treated worms were exposed to 150 and 250 µM juglone for 3.5 h at 20°C and then scored for viability. The test was performed three times. Error bars represent the standard error, and differences compared to the control (0 µg/mL, 0.1% DMSO) were considered significant at *p*<0.05 (*), *p*<0.01 (**) by one-way ANOVA and the LSD post-hoc test. ns, no significant.
(TIF)
