# Supplementary figures and images for: Monascus-Fermented Dioscorea Enhances Oxidative Stress Resistance via DAF-16/FOXO in Caenorhabditis elegans
Source: PLoS One. 2012 Jun 22;7(6):e39515. doi: 10.1371/journal.pone.0039515 (PMC3382167; doi:10.1371/journal.pone.0039515)

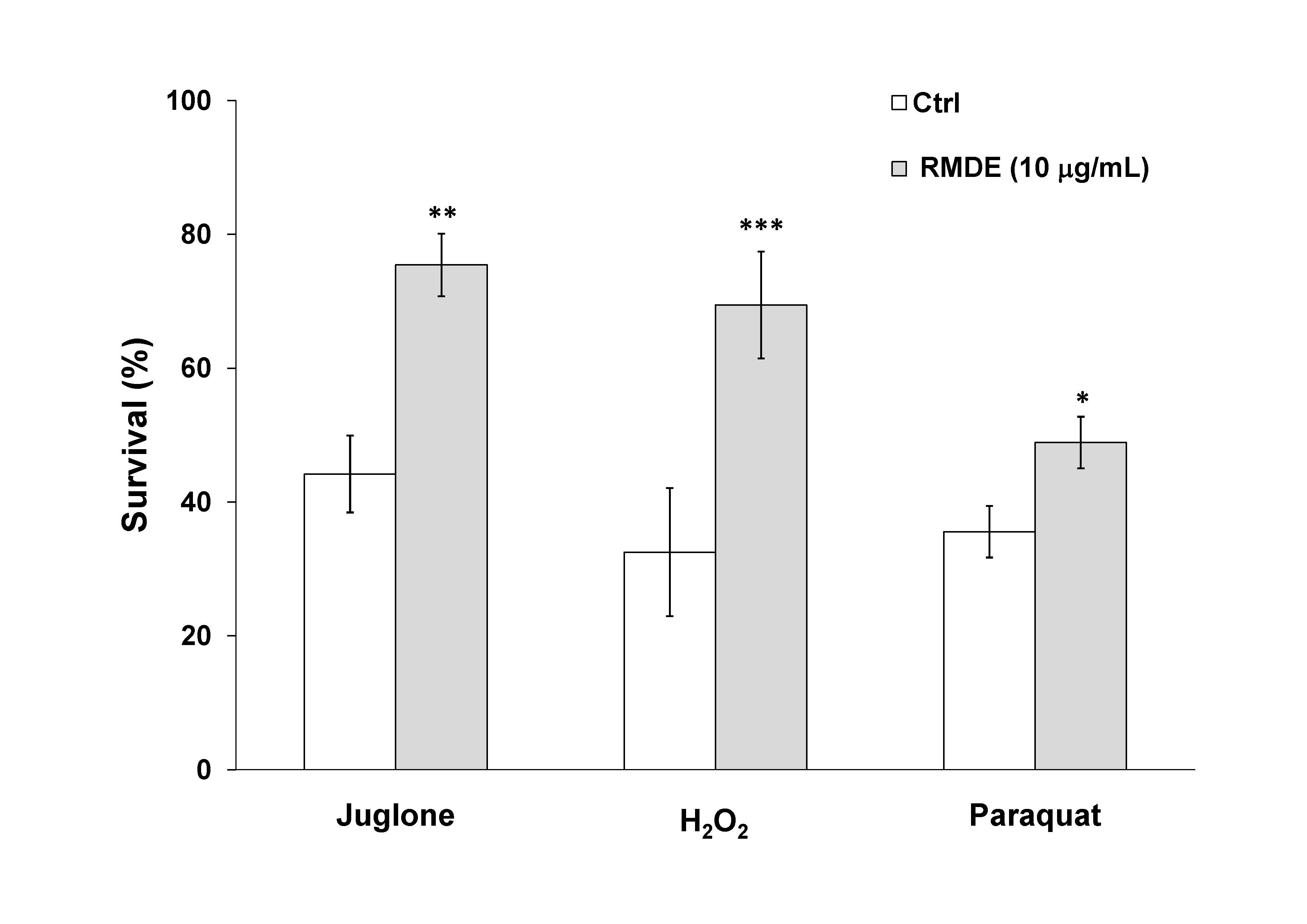

Supplement: Figure S1 — Effects of the ethanol extract of red mold dioscorea (RMDE) on oxidative-stress resistance of wild-type (WT) Caenorhabditis elegans N2 using different oxidative stress agents. Synchronized WT L1 larvae were pretreated with the RMDE 10 µg/mL or 0.1% DMSO as the solvent control for 72 h at 20°C. Subsequently, adult worms were subjected to oxidative-stress assays. Worms used for oxidative-stress assays were: RMDE-treated (juglone, n = 100; H2O2, n = 75; paraquat, n = 45) and 0.1% DMSO control (juglone, n = 100; H2O2, n = 75; paraquat, n = 45). Adult worms were exposed to 250 µM juglone, 1 mM H2O2, and 150 mM paraquat for 3.5 h, 4.5 h, and 12 h at 20°C, respectively and then scored for viability. The test was performed three times. Error bars represent the standard error, and differences compared to the control (0 µg/mL, 0.1% DMSO) were considered significant at p<0.05 (*), p<0.01 (**) by one-way ANOVA and the LSD post-hoc test. (TIF) [file pone.0039515.s001.tif]

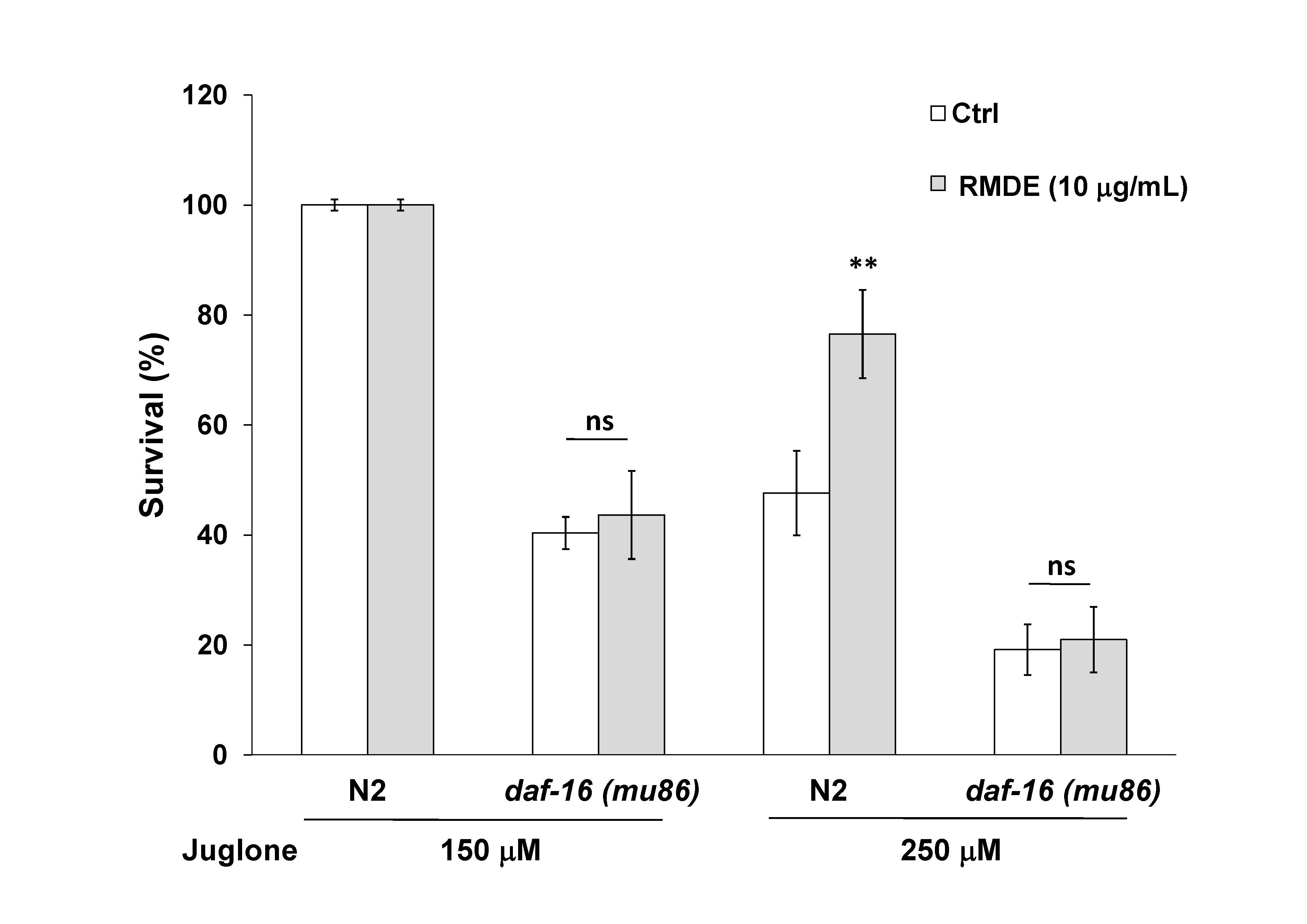

Supplement: Figure S2 — Effects of the ethanol extract of red mold dioscorea (RMDE) on DAF-16 by using different concentrations of juglone. Synchronized daf-16 (mu86) mutant L1 larvae were pretreated with the RMDE (10 µg/mL) (150 µM juglone, n = 116; 250 µM juglone, n = 118) or 0.1% DMSO as the solvent control (150 µM juglone, n = 111; 250 µM juglone, n = 143) for 72 h at 20°C. Subsequently, worms were subjected to oxidative-stress assays. RMDE-treated worms were exposed to 150 and 250 µM juglone for 3.5 h at 20°C and then scored for viability. The test was performed three times. Error bars represent the standard error, and differences compared to the control (0 µg/mL, 0.1% DMSO) were considered significant at p<0.05 (*), p<0.01 (**) by one-way ANOVA and the LSD post-hoc test. ns, no significant. (TIF) [file pone.0039515.s002.tif]
